# Assessment of rehabilitation following subarachnoid haemorrhage in China: findings from the Chinese Stroke Center Alliance

**DOI:** 10.1186/s12883-023-03349-6

**Published:** 2023-08-05

**Authors:** Yi-Tong Chen, Mei-Ru Wu, Zi-Xiao Li, Hong-Qiu Gu, Qi Zhou, Dan-Dan Wang, Yong-Jun Wang, Xing-Quan Zhao

**Affiliations:** 1 Nursing Department, Beijing Tiantan Hospital, Capital Medical University, Beijing, China; 2 Department of Neurology, Vascular Neurology Unit, Beijing Tiantan Hospital, Capital Medical University, Beijing, China; 3 China National Clinical Research Center for Neurological Diseases, Beijing, China; 4 Research Unit of Artificial Intelligence in Cerebrovascular Disease, Chinese Academy of Medical Sciences, Beijing, China; 5 Department of Neurology, Fengtai District, National Clinical Research Center for Neurological Diseases, Beijing Tiantan Hospital, Capital Medical University, No. 119 South 4Th Ring West Rd, Beijing, 100070 China

**Keywords:** Subarachnoid hemorrhage, Assessment for rehabilitation, Outcomes

## Abstract

**Background:**

Rehabilitation improves functional recovery in subarachnoid hemorrhage (SAH) patients, and assessing patients for rehabilitation is the first step in this process. However, little is known about clinical practice in China regarding the assessment and provision of rehabilitation for patients with SAH.

**Methods:**

To identify patients hospitalized with SAH and to analyze rehabilitation assessment rates, we used data for 11,234 SAH patients admitted to 861 hospitals from the China Stroke Center Alliance from August 2015 to July 2019. We examined factors for rehabilitation assessment and analyzed the relationship between rehabilitation assessment and outcomes in these patients.

**Results:**

Among 11,234 patients with SAH, 6,513 (58.0%) were assessed for rehabilitation. Assessed patients had an increased length of stay (mean ± SD days: 17.3 ± 12.5 versus 11.6 ± 10.5, P = 49.4), a higher Glasgow Coma Scale (GCS) score on admission (mean ± SD GCS score: 12.3 ± 3.8 versus 11.8 ± 4.4, P = 12.2), and were more likely to be admitted to the stroke unit (19.6% versus 13.8%, P = 15.6). In multivariable analysis, factors associated with an increased likelihood of a rehabilitation assessment (p < 0.05) included a longer length of stay (odds ratio [OR], 1.04; 95% confidence interval (CI), 1.04 to 1.05) and care such as dysphagia screening (OR, 1.88; 95% CI, 1.73 to 2.04), DVT prophylaxis (OR, 1.56; 95% CI, 1.41 to 1.72) and vessel evaluation (OR, 1.80; 95% CI, 1.63 to 1.98). For the multivariate analysis of outcomes, patients undergoing rehabilitation assessment had a longer length of stay (OR, 1.96; 95% CI, 1.81 to 2.12), a higher modified Rankin Scale (mRS) score at discharge (OR, 1.49; 95% CI, 1.36 to 1.64), and higher rates of discharge to a rehabilitation center (OR, 3.23; 95% CI, 1.81-5.75).

**Conclusion:**

More than two-fifths of SAH patients were not assessed for rehabilitation. Rates vary considerably among hospital grades, and there is a need to improve adherence to recommended care for SAH patients.

## Introduction

Stroke is a leading cause of death and disability globally, and stroke rehabilitation improves functional recovery in these patients [1]. Approximately 5% of all strokes are classified as subarachnoid hemorrhages (SAHs). A majority (85%) of non-traumatic SAHs are caused by ruptured aneurysms, with 10% due to non-aneurysmal perimesencephalic hemorrhage and the remaining 5% having rarer causes [2]. SAH is a serious and complex condition, and the 30-day mortality rate is 18% ~ 40% [3, 4]. In most countries, the incidence rate ranges from 4 to 10 per 100 000 person-years [5].

Early rehabilitation has been established as an integral part of treatment for SAH care, resulting in reduced mortality, fewer complications and better functional outcomes [5–7]. There is substantial evidence that SAH therapy is effective when provided intensively; thus, the guidelines for the clinical management of rehabilitation for subarachnoid hemorrhage recommend that some aspects of rehabilitation are similar in patients with subarachnoid hemorrhage and those with other types of stroke, patients with mild to moderate stroke can carry out bedside rehabilitation 24 h after onset, and the intensity of rehabilitation training should be individualized, giving full consideration to the physical strength, endurance and cardiopulmonary function of patients. If conditions permit, rehabilitation training of at least 45 min a day in the initial stage can improve the function of patients [8, 9]. However, as some patients require a longer time to achieve functional improvement, the time to achieve “stable” conditions differs for each SAH patient [10, 11]. Furthermore, the influencing factors of subarachnoid hemorrhage rehabilitation need to be studied based on multicenter and large samples. In China, there are few reports to date of national rehabilitation data. Therefore, it is necessary to provide data on the implementation rate and influencing factors of rehabilitation for hospitalized patients with subarachnoid hemorrhage in China.

We used data from the China Stroke Center Alliance (CSCA). The purpose of the study was to clarify the status of the rehabilitation rate and to evaluate patients with SAH to provide evidence for clinical rehabilitation care.

### CSCA design and site selection

The CSCA program is designed to establish a continuous national stroke registry and help health care providers develop stroke centers and treat patients in a consistent manner in accordance with accepted national guidelines and, ultimately, improve patient outcomes. The CSCA was designed from the CSA under the guidance of the National Center of Neurological Diseases Care Management. The CSCA is a national, hospital-based, multicenter, voluntary, multifaceted intervention and continuous quality improvement initiative. This program is available to all Chinese secondary and tertiary grade hospitals. Hospitals continued to join the program in a staggered manner. Hospital characteristics, including geographic region, teaching status, volume (secondary and tertiary grade) and annual stroke volume, were surveyed. Hospitals intending to join this program contacted the staff of CSCA voluntarily; hospitals were also recruited directly through work with the National and/or Provincial Center of Neurological Diseases Care Management. The number of hospitals from each province was obtained from the 2016 Statistical Yearbook published by the National Health and Family Planning Commission of the People’s Republic of China [12].

### Data collection and management

Data were collected via a web-based patient data collection and management tool (Medicine Innovation Research Center, Beijing, China), abstracted via chart review, coded, deidentified and transmitted in a secure manner to maintain patient confidentiality compliant with national privacy standards. The following data were collected for each hospitalized patient: demographics, history of disease and medication, hospital presentation, initial neurological status, medications and interventions, reperfusion strategy and in-hospital outcomes and complications. This patient data collection and management tool has two main functions. The first function is to collect concurrent data. The required items were structured such that valid data must be entered before the data can be saved as a complete record and submitted to the database. The second function of the tool is to analyse and provide data feedback. All hospitals using it received an independent account and password to view the benchmark for adherence to evidence-based performance measures and to compare their own hospital’s current performance to past levels as well as the concurrent standards of other regional hospitals within their purview. Information on stroke care quality was also sent to hospital personnel via the WeChat app, a communication tool for data collection and management [13].

### Outcome: assessment for rehabilitation

Assessing patients with SAH for the need for rehabilitation is an evidence-based clinical guideline and considered a core component of rehabilitation care [14, 15]. As the outcome for this study, evidence in the medical record of an “assessment for rehabilitation” was defined as follows: by documentation of (1) an assessment by members of the rehabilitation team; (2) receipt of rehabilitation services during hospitalization; (3) transfer to a rehabilitation facility; or (4) referral to rehabilitation services after discharge [16].

### Statistical analysis

The proportion of patients who received assessment for rehabilitation is described at the patient level. The adherence rate of rehabilitation assessment at the hospital level was defined as the total number of eligible patients receiving rehabilitation assessment divided by the total number of eligible patients at each hospital. Continuous variables are described as the mean ± standard deviation or median [25th-75th percentiles]. Categorical variables are presented as absolute numbers with percentages. Due to the large sample size, differences that were statistically significant may not be clinically meaningful. We used absolute standardized differences (ASDs) to compare the differences in patients’ baseline characteristics because unlike the t test, χ2 test, and other statistical tests, the ASD method is not dependent on sample size; an ASD > 10 indicated significant differences between groups [17]. Multivariable logistic regression models were performed using the backward method to identify patient characteristics as independent predictors associated with the assessment of rehabilitation and outcomes. All tests were two-tailed, and P < 0.05 was considered statistically significant. All statistical analyses were performed by using SAS (SAS Institute, NC, USA), version 9.4.

### Patient privacy and informed consent issues

Since the main purpose of the CSCA is to facilitate quality improvement, at the local level, data collection by the site is seen as a quality improvement tool. The participating hospitals are authorized to conduct quality of care assessments and have research approval to collect data from CSCA without the need for individual patient informed consent under common rules or authorization and exemption from institutional review boards. Indeed, informed consent may lead to sampling bias, which may compromise the validity and generalizability of the database. Patient confidentiality is protected in the following ways: (1) data are removed from all identifiers before their use in research, and (2) the use of data for these purposes is closely supervised by the China National Clinical Research Center for Neurological Diseases (NCRCND) analytic center [13].

## Results

### Case enrollment and target population

Patients who met the following criteria were recruited consecutively at all 1476 hospitals from August 2015 to July 2019: (1) aged 18 years or older; (2) a primary diagnosis of SAH confirmed by brain CT or MRI; (3) symptom onset within the last 7 days; and (4) admitted either directly to wards or through the emergency department. Patients with ischemic stroke, TIA, cerebral hemorrhage, cerebral venous sinus thrombosis, or non-cerebrovascular disease and patients referred to two or more hospitals within 7 days were excluded.

**Fig. 1:**
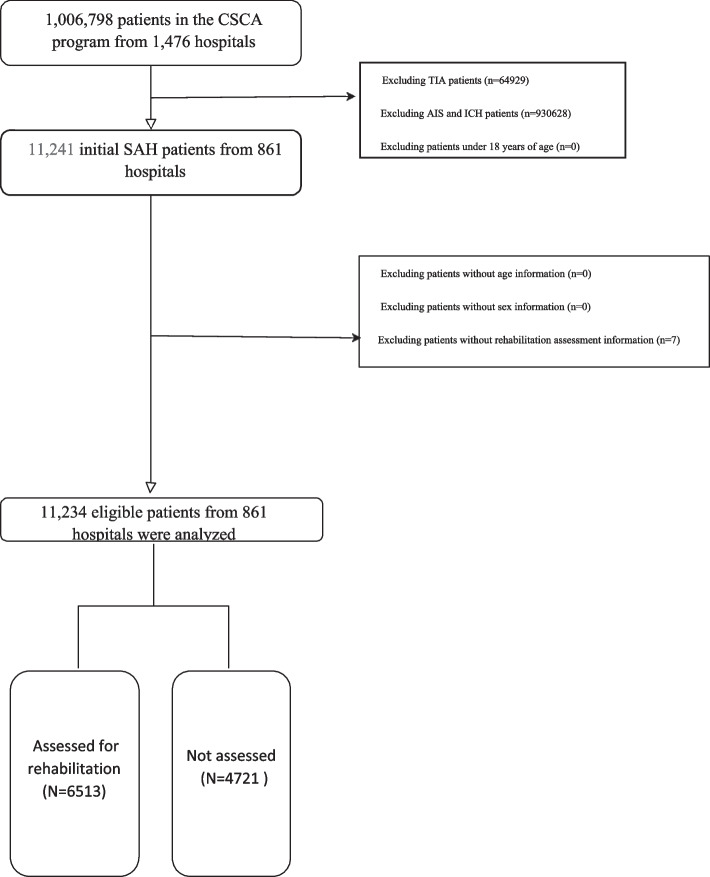
Patient selection flowchart

A total of 1,006,798 patients with acute cerebrovascular events were recruited, of whom 11,241 (1.12%) were diagnosed with SAH on admission and eligible for this study. Patients without complete rehabilitation assessment information were excluded (*N* = 7, 0.06%). The final study sample was 11,234 SAH patients from 861 hospitals (Fig. 1).

### Assessment for rehabilitation at the hospital level

**Fig. 2:**
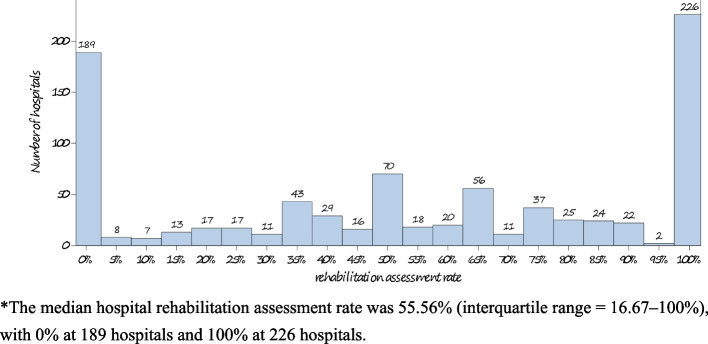
Rate of rehabilitation assessment among SAH patients by CSCA hospital

The median rate of rehabilitation assessment was 55.56% (interquartile range [IQR] 16.67–100%) and varied significantly among the 861 participating hospitals, with 0% for 189 and 100% for 226 (Fig. 2).

### Assessment for rehabilitation at the patient level

Overall, 58.0% of patients (*N* = 6,513) underwent an assessment for rehabilitation (Table 1). There were no differences between patients with or without a rehabilitation assessment in terms of age, sex, race, or medical history. When compared with patients not assessed for rehabilitation, those assessed for rehabilitation had an increased length of stay (mean ± SD day: 17.3 ± 12.5 versus 11.6 ± 10.5), a higher Glasgow Coma Scale (GCS) score on admission (mean ± SD GCS score: 12.3 ± 3.8 versus 11.8 ± 4.4), were more likely to be admitted to the stroke unit (19.6% versus 13.8%), were less likely to be an inpatient at a central hospital (39% versus 46%), and were more likely to be treated at higher grade hospital (73.4% versus 62.3%) (Table 1).

**Table 1:** Univariate analysis of SAH patient characteristics associated with documentation of rehabilitation assessment applied during hospitalization

Variables	Total (*N* = 11,234)	Assessed for rehabilitation (*N* = 6513)	Not Assessed (*N* = 4721)	ASD/HL estimator
**Demographics**				
**Age** **(y, mean** ± SD**)**	60.0 ± 12.9	59.9 ± 12.7	60.2 ± 13.3	2.3
**Sex,** Male**(n, %)**	4595 (40.9)	2675 (41.1)	1920 (40.7)	0.8
**Race,** Han**(n, %)**	10,690(95.2)	6232 (95.7)	4458 (94.4)	6.0
**BMI(n, %)**				
< 18.5	905 (8.1)	516 (7.9)	389 (8.2)	1.1
18.5–25	7572 (67.4)	4428 (68.0)	3144 (66.6)	3.0
> = 25	2757 (24.5)	1569 (24.1)	1188 (25.2)	2.6
**Insurance status(n, %)**				
NRCMS	5967 (53.1)	3409 (52.3)	2558 (54.2)	3.8
Other	564 (5.0)	310 (4.8)	254 (5.4)	2.7
Self-pa	1036 (9.2)	601 (9.2)	435 (9.2)	0.0
UEBMI	1944 (17.3)	1159 (17.8)	785 (16.6)	3.2
URBMI	1723 (15.3)	1034 (15.9)	689 (14.6)	3.6
**Education level(n, %)**				
College	426 (3.8)	247 (3.8)	179 (3.8)	0.0
High school	3344 (29.8)	1978 (30.4)	1366 (28.9)	3.3
Below Elementary	3745 (33.3)	2187 (33.6)	1558 (33.0)	1.3
Unclear	3719 (33.1)	2101 (32.3)	1618 (34.3)	4.2
** Arrival by EMS (n, %**	4681 (41.7)	2674 (41.1)	2007 (42.5)	2.8
**Hospital length**				
Nmiss (%)	112 (1.0)	72 (1.1)	40 (0.8)	
Mean ± SD	14.9 ± 12.0	17.3 ± 12.5	11.6 ± 10.5	49.4
**Admission GCS score**				
Nmiss (%)	4858 (43.2)	2694 (41.4)	2164 (45.8)	
Mean ± SD	12.1 ± 4.0	12.3 ± 3.8	11.8 ± 4.4	12.2
**Admission location(n, %)**				
Stroke unit	1928 (17.2)	1276 (19.6)	652 (13.8)	15.6
NICU + ICU	2557 (22.8)	1400 (21.5)	1157 (24.5)	7.1
Other	6749 (60.1)	3837 (58.9)	2912 (61.7)	5.7
**Medical history(n, %)**				
** Previous stroke/TIA**	2339 (20.8)	1395 (21.4)	944 (20.0)	3.5
** Hypertension**	5580 (49.7)	3266 (50.1)	2314 (49.0)	2.2
** Diabetes**	749 (6.7)	438 (6.7)	311 (6.6)	0.4
** Dyslipidaemia**	350 (3.1)	213 (3.3)	137 (2.9)	2.3
** CHD/previous MI**	565 (5.0)	308 (4.7)	257 (5.4)	3.2
** Atrial fibrillation/flutter**	111 (1.0)	72 (1.1)	39 (0.8)	3.1
** Carotid stenosis**	41 (0.4)	21 (0.3)	20 (0.4)	1.7
** PVD**	97 (0.9)	73 (1.1)	24 (0.5)	6.7
** Heart failure**	41 (0.4)	23 (0.4)	18 (0.4)	0.0
** Dementia**	14 (0.1)	11 (0.2)	3 (0.1)	2.6
** Smoking**	2414(21.5)	1398(21.5)	1016(21.5)	0.0
** Alcoholism**	1808 (16.1)	1074 (16.5)	734 (15.5)	2.7
**Care delivery(n, %)**				
** Dysphagia screening**	7089 (63.1)	4583 (70.4)	2506 (53.1)	36.2
** DVT prophylaxis**	2608 (23.2)	1771 (27.2)	837 (17.7)	22.9
** Vessel evaluation**	8427 (75.0)	5377 (82.6)	3050 (64.6)	41.7
**Hospital characteristics (n, %)**				
**Region**				
East	3979 (35.4)	2384 (36.6)	1595 (33.8)	5.9
Central	4714 (42.0)	2542 (39.0)	2172 (46.0)	14.2
West	2541 (22.6)	1587 (24.4)	954 (20.2)	10.1
**Hospital grade**				
Secondary hospital	3512 (31.3)	1734 (26.6)	1778 (37.7)	23.9
Tertiary hospital	7722 (68.7)	4779 (73.4)	2943 (62.3)	23.9

### Predictors of assessment for rehabilitation

In the multivariable model, factors associated with an increased likelihood of assessment for rehabilitation included a longer length of stay (OR, 1.04; 95% CI, 1.04 to 1.05) and care such as dysphagia screening (OR, 1.88; 95% CI, 1.73 to 2.04), DVT prophylaxis (OR, 1.56; 95% CI, 1.41 to 1.72) and vessel evaluation (OR, 1.80; 95% CI, 1.63 to 1.98). In contrast, hospitalization in the ICU (OR, 0.71; 95% CI, 0.63 to 0.82), being an inpatient at a central hospital (OR, 0.8; 95% CI, 0.72–0.89), and treatment at a lower-grade hospital (OR, 0.7; 95% CI, 0.64–0.76) were associated with a lower likelihood of a rehabilitation assessment (Table 2, first column). To further explore the relationship between the degree of the initial level of consciousness and rehabilitation assessment, we carried out an additional sensitivity multivariable analysis for 6381 patients from 861 hospitals with a recorded GCS score (Table 2, second column). However, there was little change in the direction or magnitude of the variables associated with the likelihood of assessment for rehabilitation, with the sole exception of the hospital region.

**Table 2:** Multivariable models of factors associated with documentation of rehabilitation assessment among SAH patients

Variable	Patients eligible for DS with or without GCS score, OR (95%CI, *P* value) *N* = 11,234		Patients eligible for DS with GCS score, OR (95%CI, *P* value) *N* = 6381	
	**OR (95% CI)**	***P*** ** value**	**OR (95% CI)**	***P*** ** value**
**Admission to stroke unit**				
NICU + ICU vs stroke unit	0.71 (0.63–0.82)	< 0.0001	0.67 (0.57–0.80)	< 0.0001
Other vs stroke unit	0.74 (0.66–0.83)	< 0.0001	0.80 (0.69–0.93)	0.0041
**Hospital length of stay**	1.04 (1.04–1.05)	< 0.0001	1.05 (1.05–1.06)	< 0.0001
**Dysphagia screening** Yes vs No	1.88 (1.73–2.04)	< 0.0001	1.70 (1.51–1.90)	< 0.0001
**DVT prophylaxis** Yes vs No	1.56 (1.41–1.72)	< 0.0001	1.62 (1.43–1.83)	< 0.0001
**Vessel evaluation**^a^ Yes vs No	1.80 (1.63–1.98)	< 0.0001	1.71 (1.5–1.95)	< 0.0001
**Hospital grade** Secondary hospital vs Tertiary hospital	0.70 (0.64–0.76)	< 0.0001	0.76 (0.68–0.86)	< 0.0001
**Region**				
East vs West	0.91 (0.81–1.01)	0.0817	––	
Central vs West	0.80 (0.72–0.89)	0.0001	––	

The first column includes all patients (including those with missing admission GCS scores); the second column is restricted to patients for whom admission GCS scores were recorded

The admission GCS score was not included due to missing data in 43.2% of cases

^a^Vessel evaluation refers to the evaluation of blood vessels in the neck and intracranial

### Outcomes of assessment for rehabilitation

When compared with patients who were not assessed for rehabilitation, those who were assessed for rehabilitation had an extended length of stay (54.9% versus 35.1%), were more likely to have an adverse prognosis (27.9% versus 21.0%), were less likely to experience in-hospital mortality (1.5% versus 5.2%), and were more likely to be discharged home (77.1% versus 62.0%) (Table 3).

**Table 3:** Univariate analysis of outcomes of SAH patients with and without rehabilitation assessment during hospitalization

Variables	Total (*N* = 11,234)	Assessed for rehabilitation (*N* = 6513)	Not Assessed (*N* = 4721)	ASD/HL estimator
**Extended length of stay**^**a**^ Length of stay > 14 days	5233 (46.6)	3578 (54.9)	1655 (35.1)	40.6
**Adverse prognosis**^**b**^ Disability at discharge mRS score (2–5)	2810 (25.0)	1817 (27.9)	993 (21.0)	16.1
**In-hospital mortality**	341 (3.0)	95 (1.5)	246 (5.2)	20.7
**Location after discharge**				
Discharge to home	7948 (70.7)	5020 (77.1)	2928 (62.0)	33.3
Discharge to rehabilitation center	109 (1.0)	95 (1.5)	14 (0.3)	12.7

^a^Extended length of stay was defined as a stay of more than 14 days

^b^Adverse prognosis was indicated by an mRS score of 2–5 at discharge

In multivariate analysis of outcomes, compared with patients without rehabilitation assessment, those who underwent rehabilitation assessment had a longer length of stay (> 14 days) (OR, 1.96; 95% CI, 1.81–2.12), a higher modified Rankin Scale (mRS) at discharge (OR, 1.49; 95% CI, 1.36–1.64), lower in-hospital mortality (OR, 0.39; 95% CI, 0.3–0.51), higher rates of discharge to home (OR, 1.40; 95% CI, 1.28–1.54) and higher rates of discharge to a rehabilitation center (OR, 3.23; 95% CI, 1.81–5.75) (Table 4, first column). Due to high rates of missing data (43.2% of cases), the initial level of consciousness with SAH presentation measured by the GCS score was not included in the primary multivariable models. Nonetheless, to further explore the relationship between outcome and rehabilitation assessment, we performed an additional sensitivity multivariable analysis for 6381 patients from 861 hospitals who had a recorded GCS score (Table 4, second column) and observed little change in the direction or magnitude of the variables associated with the likelihood of assessment.

**Table 4:** Multivariable models of factors associated with outcomes of SAH patients with and without rehabilitation assessment during hospitalization

**Variable**	**Patients eligible for DS with or without GCS score, OR (95%CI** **, ** ***P*** value)* ***N*** ** = 11,234**		**Patients eligible for DS with GCS score, OR (95%CI** **, ** ***P*** ** value)**# ***N*** ** = 6381**	
**Length of stay > 14 days**	1.96 (1.81–2.12)	< 0.0001**	2.17 (1.95–2.42)	< 0.0001^##^
**Disability at discharge-mRS score (2–5)**	1.49 (1.36–1.64)	< 0.0001	1.84 (1.62–2.09)	< 0.0001
**In-hospital mortality**	0.39 (0.3–0.51)	< 0.0001	0.44 (0.32–0.62)	< 0.0001
**Discharge to home**	1.40 (1.28–1.54)	< 0.0001	1.24 (1.1–1.41)	0.0007
**Discharge to rehabilitation centre**	3.23 (1.81–5.75)	0.0001	6.39 (2.28–17.93)	0.0004

The first column includes all patients (including those with missing admission GCS scores); the second column is restricted to patients for whom admission GCS scores were recorded

^*^adjusted variables: Hospital length of stay, Region, Hospital grade, Admission to the Stroke unit, Dysphagia screening, DVT prophylaxis, Vessel evaluation; **adjusted variables: Region, Hospital grade, Admission to the stroke unit, Dysphagia screening, DVT prophylaxis, Vessel evaluation; ^#^(Sensitivity analysis): adjusted variables: GCS score, Hospital length of stay, Region, Hospital grade, Admission to the stroke unit, Dysphagia screening, DVT prophylaxis, Vessel evaluation; ^##^(Sensitivity analysis): adjusted variables: GCS score, Region, Hospital grade, Admission to the stroke unit, Dysphagia screening, DVT prophylaxis, Vessel evaluation

## Discussion

Assessment for the rehabilitation of SAH patients is an evidence-based recommendation [18] and performance measure that reflects the quality of stroke care [19]. In this study of 861 hospitals in China, we observed much room for improvement in the assessment of rehabilitation. Despite national guidelines, only 58% of SAH patients were assessed for rehabilitation. These numbers are lower than the unfavorable data for rehabilitation assessment documentation from other studies. For example, in Australia, 63% ~ 90% of stroke patients were found to have been assessed by a hospital clinician or rehabilitation specialist [20, 21]. In contrast, 97.4% of stroke patients accepted rehabilitation assessment in the Get With The Guidelines-Stroke registry in the United States [22]. Moreover, the rate of rehabilitation assessment at the hospital level is differentiated in CSCA, with one-fifth of hospitals never performing rehabilitation assessment for patients with SAH and one-fourth of performing rehabilitation assessment for all patients with SAH. This reflects the unbalanced emphasis on rehabilitation assessment for SAH patients in hospitals across China, leading to serious polarization in the rate of assessment. Our findings support a continued emphasis on the need to improve the overall quality of stroke rehabilitation.

In this study, compared with patients admitted to the stroke unit, patients admitted to the ICU or other departments had significantly lower rates of rehabilitation assessment. According to the findings of evidence-based medicine, only the stroke unit (OR, 0.71) is considered the most effective site of treatment for stroke [23], comprising not a drug or a technique but a new model of ward management. The work that occurs in the stroke unit involves a diversified medical model, and the basic work mode is the teamwork mode of the stroke group. Indeed, the stroke unit carries out the overall management of patients during their hospitalization, and patients should also receive rehabilitation and health education in addition to drug treatment [24]. According to guidelines from around the world, it is recommended that all patients receive early assessment and begin rehabilitation in the stroke unit. This is because early and coordinated rehabilitation is one of the main factors contributing to the positive effects of stroke units [21]. Therefore, patients hospitalized in the stroke unit are more likely to receive rehabilitation assessments.

The median hospital length of stay of SAH patients in our study was 14 days, and the longer the hospital stay was, the more likely a patient was to receive rehabilitation assessment. This finding is similar to the report of the Get With The Guidelines program in America that a shorter length of stay is associated with a lower chance of receiving an assessment for rehabilitation [16]. In both Australia and Norway, the median hospital length of stay for acute stroke in recent years has been five days. The rehabilitation of acute inpatient cases is usually carried out in separate wards or in hospitals that provide care through inpatient rehabilitation facilities, and decisions regarding rehabilitation need to be made early [21, 25]. Moreover, recent research has shown that approximately 50% of surviving SAH patients have different levels of injury-related neurological impairment and persistent physical, social, emotional, and cognitive difficulties that prevent return to independent life and work [5, 26]. In general, the longer is the hospital length of stay of SAH patients, the more severe their condition is and the more severe their physical and cognitive impairment is; thus, they are more likely to be assessed for rehabilitation.

The quality of medical care is receiving increasing attention worldwide [27]. We found that patients who received delivery of care including dysphagia screening, DVT prophylaxis and vessel evaluation were more likely to receive a rehabilitation assessment than were patients who did not receive such care. Some SAH patients exhibit clear signs of a need for rehabilitation, such as hemiplegia and dysphagia. This study shows that patients who do not undergo rehabilitation assessment have lower GCS scores than those who do, because patients with low GCS scores have serious impairment of consciousness and are unable to cooperate with rehabilitation assessment programs such as muscle strength tests. Furthermore, patients with a low GCS score on admission (impaired consciousness and severe illness) visually exhibit a need for rehabilitation even without formal assessment. They may also be identified as needing dysphagia screening, DVT prevention, or limb placement early during hospitalization. In China, pneumatic pressure devices and good limb placement are mainly carried out by nurses. Each of these features increases the likelihood of a rehabilitation assessment.

Compared with secondary hospitals, tertiary hospitals have rich medical resources and treat patients with complicated conditions. Overall, the medical personnel at these facilities carry out more comprehensive treatment and intervention, pay more attention to the prognosis of SAH patients and are more likely to perform rehabilitation assessments. Although higher grade hospitals are more likely to assess SAH patients for rehabilitation, a greater number of stroke discharges annually decrease the likelihood of assessment. The total amount of medical resources per capita is low in China compared with most developed countries [28]. For example, the rate per capita of beds and doctors in China per 1000 persons is 4.85 and 2.12 (2014) compared with 2.93 and 2.56 (2012) in the United States, respectively, and bed occupancy rates in China are 90.1% compared with 64.4% in the US [29]. As rehabilitation resources tend to be fixed, the annual increase in stroke discharges indicates that the per capita rehabilitation capacity is insufficient. Moreover, 80% of medical beds are allocated in urban areas, and 80% of the beds in cities are allocated to large hospitals, which leads to a very low efficiency in the macro-allocation of medical and health resources in China [29]. Uneven distribution of limited medical resources and increased demand may lead to a reduced likelihood of rehabilitation assessments. Other possible reasons include a patient's greater willingness to return home or to transfer to a lower-level hospital before rehabilitation assessment due to their condition. In addition to increasing the number of rehabilitation professionals in each hospital, multidisciplinary teamwork and remote consultation may increase the chance of assessment and rehabilitation services before discharge.

Compared with patients who did not undergo rehabilitation assessment, those with rehabilitation assessment had a longer length of stay, higher levels of disability (mRS scores) at discharge, lower in-hospital mortality, were more likely to be discharged to home and were more likely to be discharged to a rehabilitation center. Some general hospitals contain independent rehabilitation wards, and SAH patients who have undergone rehabilitation assessment can be rehabilitated in their own hospital if they need rehabilitation training without transfer, thus increasing the length of stay. In contrast, some lower-level hospitals do not have rehabilitation facilities, and therefore patients need to be transferred to rehabilitation centers for further rehabilitation training after rehabilitation assessment. Patients who return home are divided into two categories: those with mild illness, no residual disability, and no rehabilitation training; and those with a particularly severe condition, near death, and with no need for rehabilitation.

### Limitations

The limitations of this study included the following. First, hospital registry participation was voluntary, and participating hospitals were more likely to focus on improvements in stroke care quality. These hospitals may represent a higher standard of stroke care than is available nationwide. Second, a high rate of missing data for the GCS score limited our ability to adjust for confounding by SAH severity. Third, this study did not exclude patients who were unsuitable for physical therapy, such as patients who died within 1–3 days after a stroke or those who were transferred from a lower-grade hospital to a higher-grade hospital. Fourth, conditions may have influenced outcomes, such as lower rates of poor outcomes and in-hospital mortality among patients undergoing rehabilitation assessments. In addition, limitations of this study include not taking into consideration the clustering effect of the hospitals, and the study design was a cross-sectional study without randomization. Despite these limitations, our study provides a comprehensive characterization of rehabilitation assessments for a large cohort of SAH patients.

## Conclusion

In summary, a documented assessment for rehabilitation was not found for two-fifths of patients with SAH, which was associated with a greater than threefold increase in mortality. Analysis of SAH patients treated at hospitals in CSCA identified that the rate of rehabilitation assessment should be improved. Establishing standards for rehabilitation in care may require local customization with additional training, consultation and multidisciplinary teamwork to match the resources available. Studies on the effects of rehabilitation in different models of SAH in China can support improvements in evidence-based recommendations for care.

## Data Availability

The datasets used and/or analysed during the current study are available from the corresponding author upon reasonable request.

## Abbreviations

BMI: Body mass index
NRCMS: New rural cooperative medical scheme
UEBMI: Urban employee basic medical insurance
URBMI: Urban resident basic medical insurance
EMS: Emergency medical services
GCS: Glasgow Coma Scale
NICU: Neurological intensive care unit
ICU: Intensive care unit
TIA: Transient ischemic attack
CHD: Coronary heart disease
MI: Myocardial infarction
PVD: Peripheral vascular disease
DVT: Deep vein thrombosis
DS: Dysphagia screening
OR: Odds ratio
CI: Confidence interval
